# CircWHSC1 Promotes Breast Cancer Progression by Regulating the FASN/AMPK/mTOR Axis Through Sponging miR-195-5p

**DOI:** 10.3389/fonc.2021.649242

**Published:** 2022-01-05

**Authors:** Qian Chen, Zhen Yang, Hongjian Ding, Huaqing Li, Weiyu Wang, Zhiyu Pan

**Affiliations:** ^1^ Department of General Surgery, Minhang Hospital, Fudan University, Shanghai, China; ^2^ Zhongnan Hospital of Wuhan University, Institute of Hepatobiliary Diseases of Wuhan University, Transplant Center of Wuhan University, Hubei Key Laboratory of Medical Technology on Transplantation, Wuhan, China

**Keywords:** sponging, FASN, MiR-195-5p, circWHSC1, breast cancer

## Abstract

Numerous studies reveal that circular RNAs (circRNAs) affect cancer progression. CircWHSC1 is a novel circRNA that accelerates ovarian cancer progression. Nevertheless, the function of circWHSC1 in regulating breast cancer (BC) is elusive. Here, quantitative reverse transcription-polymerase chain reaction (qRT-PCR) was carried out to detect the profiles of circWHSC1 and miR-195-5p in BC tissues and corresponding non-tumor tissues. Gain- and loss-of-function assays were implemented both *in vivo* and *ex vivo* to verify the significance of circWHSC1 in BC development. BC cell proliferation was estimated by the cell counting kit-8 (CCK-8) and BrdU assays. Transwell assay was implemented to test BC cell migration and invasion. The protein levels of FASN, AMPK and mTOR were determined by Western blot. Moreover, immunohistochemistry was performed to examine Ki67 and FASN expression. As shown by the result, circWHSC1 was up-regulated in BC tissues versus adjacent non-tumor tissues. circWHSC1 overexpression was correlated with higher tumor stages, lymphatic metastasis and worse survival of BC patients. Functionally, overexpressing circWHSC1 amplified proliferation, migration and invasion of BC cell lines and boosted xenograft tumor growth in nude mice. Bioinformatics uncovered that circWHSC1 functioned as a competitive endogenous RNA by sponging miR-195-5p, which was further corroborated by the dual-luciferase reporter assay and RNA immunoprecipitation. miR-195-5p delayed BC progression, which was dampened by circWHSC1 up-regulation. Fatty acid synthase (FASN) was affirmed as a direct target of miR-195-5p. miR-195-5p overexpression curbed FASN expression and activated its downstream AMPK pathway. Inhibition of FASN or activation of the AMPK pathway reversed circWHSC1-mediated oncogenic effects. Collectively, CircWHSC1 acted as an oncogene to expedite BC evolvement by modulating the miR-195-5p/FASN/AMPK/mTOR pathway.

## Introduction

Breast cancer (BC) is a familiar malignancy harming women’s health worldwide (1). Hormone therapy is only appropriate for HR-positive cancer patients. Surgery is usually suitable for early BC patients or those who have received neoadjuvant treatment instead of metastatic patients. Thus, the overall morbidity of BC patients is still growing due to limited methods in treating metastatic BC or triple-negative breast cancer (TNBC) (2, 3). Hence, elucidating the molecular mechanism of BC progression is vital for improving the efficacy and prognosis of BC patients.

Circular RNAs (circRNAs) are long noncoding endogenous RNAs with limited protein-coding abilities and are involved in various transcriptional and post-transcriptional genomic regulations (4). Accumulating reports have stated that circRNAs participate in tumorigenesis and tumor evolvement (5). For example, circ-0001742 regulates the miR-431-5p/ATF3 axis by functioning as a competitive endogenous RNA (ceRNA), thereby aggravating tongue squamous cell carcinoma (TSCC) progression (6). circ-0000267 promotes the proliferation, migration, invasion and epithelial-mesenchymal transition (EMT) of gastric cancer cells by up-regulating the miR-503-5p/HMGA2 axis (7). In contrast, circCDYL represses colon cancer cells’ growth and migration by inhibiting miR-150-5p (8). CircWHSC1 is a newly discovered circRNA. Overexpressing circWHSC1 inhibits miR-145 and miR-1182, and aggravates the growth and metastasis of ovarian cancer cells (9). Yet, there are few reports on the function of circWHSC1 in BC.

MicroRNAs (miRNAs) are noncoding RNAs with 20-24 nucleotides in length, which modulate gene expression through diverse mechanisms (10). miRNAs modulate cell growth and differentiation, act as promising tumor markers and contribute to cancer development. For example, miR-424-5p is a contributor of the proliferation, migration, invasion and adhesion of laryngeal squamous cell carcinoma (LSCC) cells by up-regulating CADM1 (11). miR-627-5p decreases the proliferation and metastasis of hepatoma cells by suppressing BCl3 (12). As an important miRNA, miR-195-5p is located at 17p13.1 and 87bp in length, which has a role in controlling cell proliferation and migration. For example, INC-ROR facilitates the proliferation and migration of aortic smooth muscle cells by regulating the miR-195-5p/FGF2 pathway (13). The mechanism by which miR-195-5p modulates BC progression is, however, unclear and this requires further study.

Fatty acid synthase (FASN) is located at 17q25.3 and is 8464bp in length. Functionally, FASN catalyzes the synthesis of acetyl CoA and malonyl CoA and palmitic acid into long saturated fatty acids in the presence of NADPH (14). Interestingly, FASN is abnormally expressed in tumor tissues and mediates tumor evolvement (15). For instance, FASN enhances EMT in ovarian cancer through transcriptional regulation of E-cadherin and N-cadherin. Meanwhile, knocking down FASN dampens ovarian cancer invasion and migration both *in vivo* and *in vitro* (16). In addition, FASN gets involved in the regulation of cancer-associated metabolic reprogramming, and targeting FASN has been regarded as an effective method in treating BC (17).

Here, we found that circWHSC1 was up-regulated in BC, and higher expression of circWHSC1 predicted worse survival of BC patients. Functionally, circWHSC1 promoted BC cells’ proliferation, migration and invasion. Moreover, we verified a targeted regulatory correlation among circWHSC1, miR-195-5p and FASN through biological information analysis. By detecting circWHSC1, miR-195-5p and FASN expressions in BC tissues and cells and probing the association between these molecules, we confirmed that circWHSC1 targets miR-195-5p by acting as a ceRNA and up-regulates FASN. In summary, this research sheds light on the molecular mechanisms in BC evolvement and presents novel references for BC therapy.

## Material and Methods

### Clinical Specimen Collection and Handling

The tumor tissues and adjacent non-tumor tissues from 50 primary BC patients who received surgery in Minhang Hospital of Fudan University from October 2014 to October 2015 were collected. No chemotherapy, radiotherapy, or other neoadjuvant treatments were performed on those patients before operation. No cancer cells were observed in the adjacent non-tumor tissues by postoperative pathological examination. All samples were isolated and promptly preserved in liquid nitrogen at -196°C for RNA extraction. The research ethics committee of Minhang Hospital of Fudan University authorized this study, and informed agreement was obtained from all participating patients.

### Cell Culture

Human normal breast epithelial cells MCF-10A and BC cell lines (MCF7, BT474, SKBr-3, ZR-75-30, MDA-MB-453 and MDA-MB-231) were bought from ATCC (Rockville, USA). These cells were grown in the RPMI-1640 complete medium (Thermo Scientific Hyclone, Utah, USA) which contained 10% fetal bovine serum (FBS, Thermo Scientific Hyclone, Utah, USA) and 1% penicillin/streptomycin (Yeasen Biotech Co., Ltd., Shanghai, China) and kept at 37°C with 5% CO_2_ and saturated humidity. The medium was replaced every 2 to 3 days. When the cells got 90% confluency, 0.25% trypsin (Thermo Scientific Hyclone, Utah, USA) was adopted for cell trypsinization and subculture.

### Cell Transfection

MCF7 and MDA-MB-231 cells in the logarithmic growth phase received trypsinization by 0.25% trypsin. The cells were collected and seeded in 6-well plates with 5×10^6^ cells per well. 24 hours later, the two cell lines were transfected with circWHSC1 overexpressing plasmids, miR-195-5p mimics and their negative controls by applying the Lipofectamine™ 3000 Transfection Reagent (Thermo, Shanghai, China) as per the manufacturer’s instructions. Afterward, the cells were kept at 37°C with 5% CO_2_. After 24 hours of transfection, the medium was exchanged with a new compete medium and the cells were kept culturing for another 24 hours. After that, the total RNA was extracted from the cells, and quantitative reverse transcription-polymerase chain reaction (qRT-PCR) was performed to detect the expressions of circWHSC1 and miR-195-5p.

### qRT-PCR

The total RNA was extracted out of cells by using the TRIzol reagent. After RNA concentration and purity measurement, the total RNA was reversely transcribed into cDNA with the PrimeScript ™ RT Reagent kit (Invitrogen, Shanghai, China). The procedures were conducted according to the manufacturer’s instructions. Subsequently, the Bio-Rad CFX96 quantitative PCR system and SYBR Green Real-Time PCR Master Mixes (Thermo Fisher Scientific, Shanghai, China) were utilized to conduct qRT-PCR. The reaction conditions included: pre-denaturation (95°C, 5 min), denaturation (95°C, 15s), and annealing (60, 30s). GAPDH acted as a housekeeping gene for calculating the relative circWHSC1 and FASN expression, and U6 was utilized as an internal control for calculating the relative miR-195-5p expression. The 2^(-ΔΔCt)^ method was employed for relative gene expression analysis. The primers were as follows: miR-195-5p Forward: 5’-AACCGGTAGCAGCACAGAAATG-3’, Reverse: 5’-CAGTGCAGGGTCCGAGGT-3’; CircWHSC1 Forward: 5’-CGATGTTTAAGCGTCCGGG-3 ‘, Reverse: 5’-GGAAAGATGATGCGCTGTGT-3’ FASN Forward: 5’-GTGAACTGCTGCACGAAGAA-3’; Reverse: 5’-GCCTTTGAAATGTGCTCCCA-3’; GAPDH Forward: 5’-TGGTTGAGCACAGGGTACTT-3’; Reverse: 5’-CCAAGGAGTAAGACCCCTGG-3’; U6: Forward: 5’- CTCGCTTCGGCAGCACA-3’; Reverse: 5’-AACGCTTCACGAATTTGCGT-3’.

### Cell Counting Kit-8 (CCK-8) Assay

MCF7 and MDA-MB-231 cells in the logarithmic growth phase were trypsinized, resuspended (2×10^3^/mL) and inoculated in 96-well plates with 100 μL cell suspension per well. Three replicate wells were established per group. The plates were then maintained in an incubator for further culture. After 24 hours, 10 μL of CCK-8 solution (Hubei Biossci Biotechnology Co., Ltd.) was added to each well, and the cells were cultured for 1 hour. After the culture was completed, the optical density (OD) value of each well at 450 nm was measured at the 24^th^, 48^th^, 72^th^, and 96^th^ hours.

### BrdU Assay

MCF7 and MDA-MB-231 cells were seeded into 24-well plates at 1×10^5^ cells/well. 24 hours later, the BrdU reagent (Sigma, Beijing, China) was added into each group, and the plates were incubated at 37°C with 5% CO_2_. After the cells were cultured for 48 hours, immunofluorescence staining was performed according to the anti-BrdU antibody (Abcam) operation guidelines. DAPI (Beyotime, Shanghai, China) was used for labeling the nucleus. The BrdU-positive cells and DAPI-positive cells were counted in 3 random areas with the fluorescence microscope (Olympus, Japan). Cell proliferation rate=the number of BrdU-positive cells/DAPI-positive cells×100%.

### Transwell Assay

MCF7 and MDA-MB-231 were seeded in 24-well culture plates with a transwell chamber (Corning, Beijing, China). The chambers were coated with Matrigel (Cat.No. 356234, BD company, Germany) in the invasion test, while they were not pre-coated with Matrigel in the migration experiment. The upper chambers were supplemented with 5×10^4^ cells. In parallel, 10% FBS medium and 400 μL of RPMI-1640 was added to the lower chamber. After an incubation at 37°C for 24 hours, non-migrated or noninvasive cells were wiped off. 4% paraformaldehyde was used for fixing the cells for 10 minutes at room temperature, and then 0.5% crystal violet was adopted for cell staining. After rinsing with running water, an inverted microscope was used for cell counting. All tests were done in triplicate.

### Dual-Luciferase Reporter Assay

All luciferase reporter vectors (CircWHSC1-WT, CircWHSC1-MT, FASN-WT, and FASN-MT) were constructed by Promega Corporation (Madison, WI, USA). CircWHSC1-MT and FASN-MT were mutant at the binding sites with miR-195-5p, whereas CircWHSC1-WT and FASN-WT were the wild type vectors containing the binding sites with miR-195-5p. MCF7 cells (4.5×10^4^) were seeded in 48-well plates. After 24-hour culture, the cells were co-transfected with CircWHSC1-WT, CircWHSC1-MT, FASN-WT, FASN-MT and miR-195-5p mimics or negative control by utilizing Lipofectamine™ 3000. Following 48 hours, the dual luciferase activities were monitored. All experiments were conducted three times.

### Western Blot (WB)

MCF7 and MDA-MB-231 cells were seeded in 6-well plates (6×10^5^/well). Seventy-two hours later, the total protein was extracted from the two cell lines using RIPA (Beyotime, Shanghai, China). The BCA method (Beyotime, Shanghai, China) was employed for protein content measurement. 20 μg total protein in each group was subjected to 10% polyacrylamide gel electrophoresis and then transferred to the PVDF membranes, which were blocked with 5% skimmed milk. Afterward, the membranes were incubated with the primary antibodies (Abcam, MA, USA), including anti-Casase3 (ab32351, 1: 1000), anti-Bax (ab32503, 1: 1000), anti-Bcl2 (ab32124, 1: 1000), anti-Vimentin (ab92547, 1: 1000), anti-E-cadherin (ab40772, 1: 1000), anti-N-cadherin (ab76011, 1: 1000), anti-FASN (ab22759, 1: 1000), anti-AMPK (ab3047, 1:1000), anti-p-AMPK (ab23875, 1: 1000), anti-mTOR (ab2732, 1: 1000) and anti-p-mTOR (ab109268, 1:1000) at 4°C overnight. Next, they were kept with the goat anti-rabbit IgG (ab150077, 1: 2500, Abcam, MA, USA) at room temperature (RT) for 2 hours. Subsequently, the ECL chemiluminescence reagent was used for protein blots exposure. ImageJ software was used for gray analysis. GAPDH acted as the internal control.

### RNA Immunoprecipitation (RIP)

RIP was carried out with the Magna RIP RNA binding protein immunoprecipitation kit (Millipore, Bedford, MA, USA). BC cells transfected with miR-195-5p mimics or miR-NC at 80% confluence were collected, then lysed using RIP lysis buffer. Then, the anti-Ago2 antibody (Abcam, Shanghai, China) was applied for Ago2 immunoprecipitation, and the immunoglobulin G (IgG) antibody served as a negative control. Immunoprecipitated RNA was separated and the enrichment of CircWHSC1 and FASN in the lysates was assessed by qRT- PCR.

### The Xenograft Tumor in Nude Mice

MCF7 and MDA-MB-231 cells transfected with circWHSC1 overexpression plasmids or negative controls were collected by 0.25% trypsin. Single-cell suspensions (3×10^7^/mL) were made using the serum-free medium. 6-week-old BALB/c-nu nude mice were used for establishing xenograft tumor model. 40 nude mice were divided into 4 groups, and 0.1 mL of cell suspension was administered subcutaneously into each mouse’s left forelimb. After the injection, the tumor sizes were calculated weekly for five weeks, with the mass of the tumor tissues derived from mice measured. The lung tissues of all nude mice were collected and stained by H&E to evaluate pulmonary metastases of BC cells.

### Immunohistochemistry (IHC)

Following paraffin embedding and sectioning (4 μM), the tumor tissue sections were dewaxed with xylene, hydrated with gradient alcohol, and treated with 3% H_2_O_2_ for 10 minutes. Microwave repair was made by applying 0.01 mol/L sodium citrate buffer (pH=6.0, 15 min), and the sections were blocked with 5% bovine serum albumin (BSA) for 20 minutes, followed by the incubation with anti-FASN (ab22759, Abcam, MA, USA) and Ki67 (ab15580, Abcam, MA, USA) overnight at 4°C. The following day, the goat-anti-rabbit IgG was added and kept at 37 °C for 60 minutes. After rinsing with PBS, DAB was used for color development. After hematoxylin re-staining, the sections underwent dehydration, permeation, sealing, and examination under a microscope (Olympus, Japan).

### Data Analysis

SPSS 20.0 (SPSS Inc., Chicago, IL, USA) was utilized for data analysis. Mean ± standard deviation (`x ± s) was used for data measurement. The multivariate comparison was conducted with one-way analysis of variance, and pairwise comparison was made by *t* test. *P <*0.05 was considered as statistical significance.

## Results

### The Clinical Importance of circWHSC1 in BC Tissues

**Figure 1:**
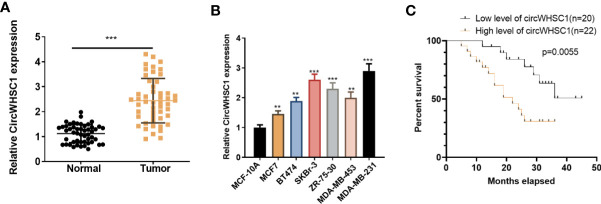
CircWHSC1 expression in non-tumor and cancer tissues. **(A)**: qRT-PCR assessed circWHSC1 profile in tumors and paired non-tumor tissues of BC patients. ***P < 0.001 (vs. Normal group). **(B)** qRT-PCR was adopted to measure the circWHSC1 profile in normal breast epithelial cells MCF-10A and BC cell lines (MCF7, BT474, SKBr-3, ZR-75-30, MDA-MB-453, and MDA-MB-231). **P < 0.01, ***P < 0.001 (vs. MCF-10A group). **(C)** Kaplan-Meier assay was used for analyzing the overall survival of BC with high or low levels of circWHSC1.

To verify the function of circWHSC1 in BC, we performed qRT-PCR. The data indicated that circWHSC1 was notably overexpressed in BC tissues compared with that in the adjacent non-tumor tissues (*P <*0.05, **Figure 1A**). Besides, circWHSC1 was distinctly up-regulated in BC cell lines (MCF7, BT474, SKBr-3, ZR-75-30, MDA-MB-453 and MDA-MB-231) by contrast with that in human normal breast epithelial cell MCF-10A (*P <*0.05, **Figure 1B**). Next, we evaluated the function of circWHSC1 in predicting the survival of BC patients. As a result, a higher level of circWHSC1 was markedly linked to worse overall survival of BC patients (**Figure 1C**). Meanwhile, BC patients with a higher expression of circWHSC1 had a more advanced tumor stage, more lymphatic metastasis and distant metastasis and higher Ki67 levels (**Table 1**). Therefore, circWHSC1 was a potential oncogene in BC.

**Table 1:** Relationship between CircWHSC1 expression level and clinical characteristics of BC patients.

Characteristics	Patients	Expression of CircWHSC1		P-value
		Low-CircWHSC1	High-CircWHSC1	
Total	50	20	30	
Age (years)				0.9074
<55	28	11	17	
≥55	22	9	13	
Tumor stage				0.0184*
I-II stage	20	12	8	
III-IV stage	30	8	22	
Lymphatic metastasis				0.028*
Positive	19	13	10	
Negative	31	7	20	
Tumor size				0.1655
<3cm	26	8	18	
>3cm	24	12	12	
Histological differentiation				0.7664
Low-grade	16	5	11	
Middle-grade	20	9	11	
High-grade	14	6	8	
Distant metastasis				0.049*
M0	23	13	11	
M1	27	7	19	
Ki-67 level (%)				0.0217*
≤10	27	14	13	
>10	23	6	17	

*P < 0.05.

### Impacts of circWHSC1 on BC Cell Proliferation, Migration and Invasion

**Figure 2:**
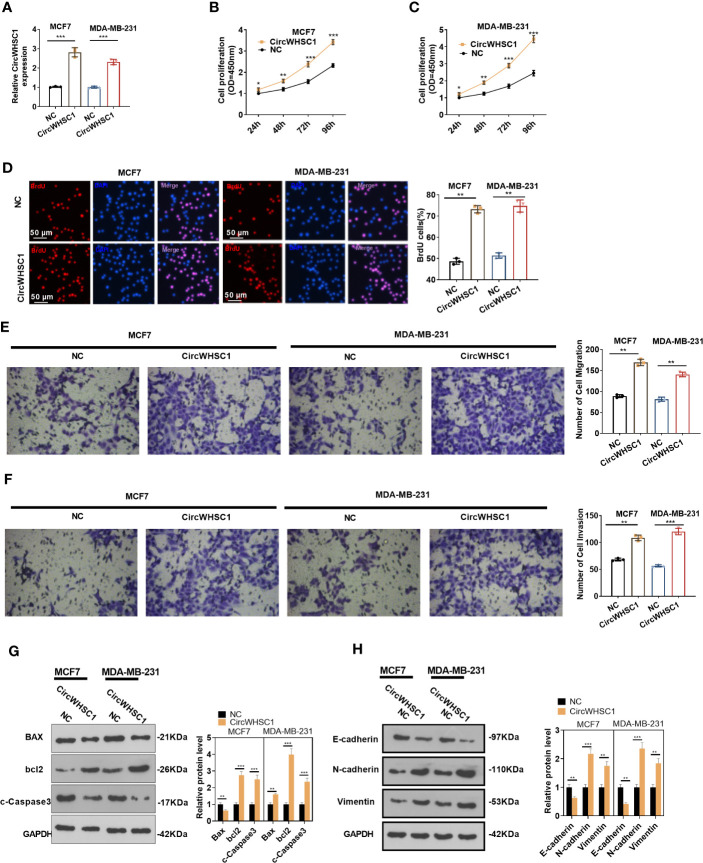
CircWHSC1 affected BC cell proliferation, migration and invasion. **(A)** CircWHSC1 overexpression models were set up in MCF7 and MDA-MB-231, respectively. **(B–D)** CCK-8 **(B, C)** and BrdU assay **(D)** detected cell viability and proliferation, *P < 0.05, **P < 0.01, ***P < 0.001 vs. NC group. scale bar=50 μm. **(E, F)** The effect of CircWHSC1 overexpression on cell migration and invasion was testified by the transwell assay. **(G, H)**. WB was performed for evaluating the protein levels of Bax, bcl2, c-Caspase3, E-cadherin, N-cadherin, and Vimentin. *P < 0.05, **P < 0.01, ***P < 0.001. N=3.

For the purpose of exploring how circWHSC1 affected BC evolvement, we constructed circWHSC1 overexpression cell models in MCF7 and MDA-MB-231 (**Figure 2A**). CCK-8 and BrdU methods demonstrated that cell proliferation was markedly facilitated after circWHSC1 overexpression versus the NC group (*P <*0.05, **Figures 2B**–**D**). Further, transwell assay was conducted to study circWHSC1’s impact on cell migration and invasion. As displayed in the figure, cell migration and invasion were dramatically enhanced after circWHSC1 overexpression (*P <*0.05, **Figures 2E, F**). WB results testified that circWHSC1 overexpression reduced BAX, c-Caspase3, and E-cadherin levels, whereas up-regulated bcl2, N-cadherin, and Vimentin (**Figures 2G, H**). Those data indicated that circWHSC1 promotes the malignant phenotypes of BC cells and plays a cancerigenic role.

### Overexpressing circWHSC1 Amplified BC Cells Growth *In Vivo*

**Figure 3:**
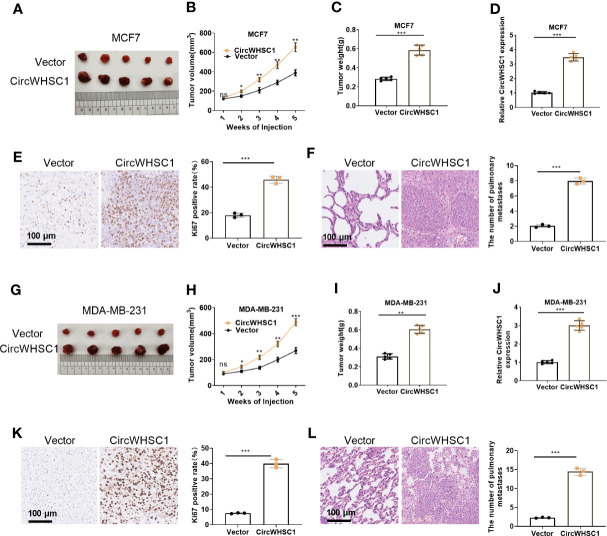
Overexpression of circWHSC1 boosted BC cell growth in vivo. MCF7 cells were transfected with vector or CircWHSC1 overexpression plasmids and then subjected to constructing subcutaneous Xenograft models on nude mice. **(A–D)** Nude mice were sacrificed 5 weeks later, and subcutaneous tumors were extracted. Tumor volume **(B)** and weight **(C)** were calculated, and the expression of circWHSC1 was estimated by qRT-PCR **(D)**. **(E)** IHC detected KI67 expression in the tumors formed by MCF7 cells. **(F)** H&E staining examined lung metastasis of MCF7 cells. MDA-MB-231 cells were transfected with vector or CircWHSC1 overexpression plasmids and then subjected to constructing subcutaneous Xenograft models on nude mice. **(G–J)** Nude mice were sacrificed after 5 weeks, and subcutaneous tumor nodules were removed. The tumor volume **(H)** and weight **(I)** were calculated, and the expression of circWHSC1 was estimated by qRT-PCR **(J)**. **(K)** IHC checked the number of KI67-positive cells in MDA-MB-231 cells. **(L)** H&E staining monitored lung metastasis of MDA-MB-231 cells. ns *P* > 0.05, *P < 0.05, **P < 0.01, ***P < 0.001 (vs.Vector group). N=5. scale bar=100 μm.

We constructed circWHSC1 overexpression models in MCF7 and MDA-MB-231 to verify its role on cell growth *in vivo*. It was found that in the MCF7 cell lines, the tumor size and mass in the circWHSC1 overexpression group were distinctly increased versus the vector group (*P <*0.05, **Figures 3A**–**D**). Immunohistochemical outcomes corroborated that the ratio of Ki67-positive cells was increased after circWHSC1 overexpression, indicating a significant increase in tumor proliferation (*P <*0.05, **Figure 3E**). At the same time, lung metastases increased (*P <*0.05, **Figure 3F**). Similar outcomes were found in MDA-MB-231 cell lines with circWHSC1 overexpression (*P <*0.05, **Figures 3G**–**L**). Thus, overexpressing circWHSC1 enhanced BC cell growth.

### CircWHSC1 Targeted miR-195-5p and Abated Its Profile

**Figure 4:**
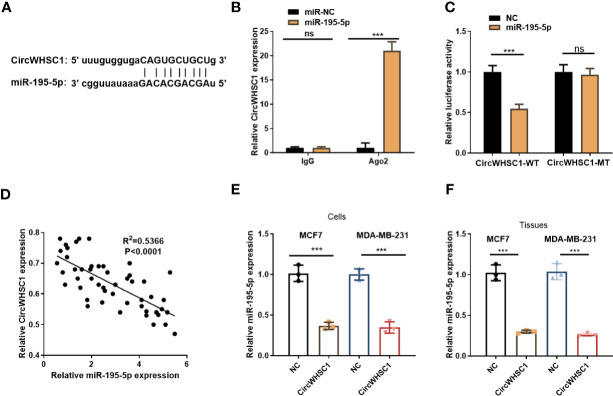
CircWHSC1 targeted and abated miR-195-5p level. **(A)** The binding sites between circWHSC1 and miR-195-5p were analyzed by Starbase. **(B)** RIP assay was performed for verifying the binding between circWHSC1 and miR-195-5p. The enrichment of circWHSC1 in the lysates was determined by qRT-PCR. **(C)** The dual-luciferase reporter gene assay verified the binding correlation between circWHSC1 and miR-195-5p. **(D)** Pearson linear regression analysis checked circWHSC1 and miR-195-5p expression in BC tissues.**(E, F)** miR-195-5p expression in MCF7 and MDA-MB-231 cells transfected with NC or circWHSC1 overexpression plasmids **(E)** or in xenograft tumors formed by MCF7 and MDA-MB-231 cells (with NC or circWHSC1 overexpression plasmids transfection) **(F)** was evaluated by qRT-PCR. ns *P* > 0.05, *P < 0.05, **P < 0.01, ***P < 0.001 (vs. NC group). N=3.

To figure out the downstream mechanism of circWHSC1, we analyzed the potential target genes of circWHSC1 *via* Starbase (http://starbase.sysu.edu.cn/) and discovered miR-195-5p was a potential target of circWHSC1 (**Figure 4A**). Next, we performed RIP in MCF7 cells. It turned out that the circWHSC1 enrichment in the Ago2 antibody group was obviously higher versus the IgG group after miR-195-5p transfection, hinting that circWHSC1 bound to Ago2 *via* miR-195-5p (*P <*0.05, **Figure 4B**). Furthermore, dual-luciferase reporter assay was performed. miR-195-5p mimics reduced the luciferase activity of circWHSC1-WT, while had little impact on circWHSC1-MT’s luciferase activity (*P*> 0.05, **Figure 4C**). These data affirmed the binding correlation between miR-195-5p and circWHSC1. What’s more, Pearson linear regression analysis corroborated that circWHSC1 was reversely associated with miR-195-5p levels in BC tissues (*R* ^2^ = 0.5366, *P <*0.0001, **Figure 4D**). Subsequently, we inquired into the influence of circWHSC1 overexpression on miR-195-5p by qRT-PCR. The result uncovered that miR-195-5p was notably down-regulated after circWHSC1 up-regulation (*P <*0.05, **Figures 4E, F**). In summary, miR-195-5p was targeted and inhibited by circWHSC1.

### circWHSC1 Targeted miR-195-5p and Suppressed miR-195-5p-Mediated Inhibitive Effects on BC Cells

**Figure 5:**
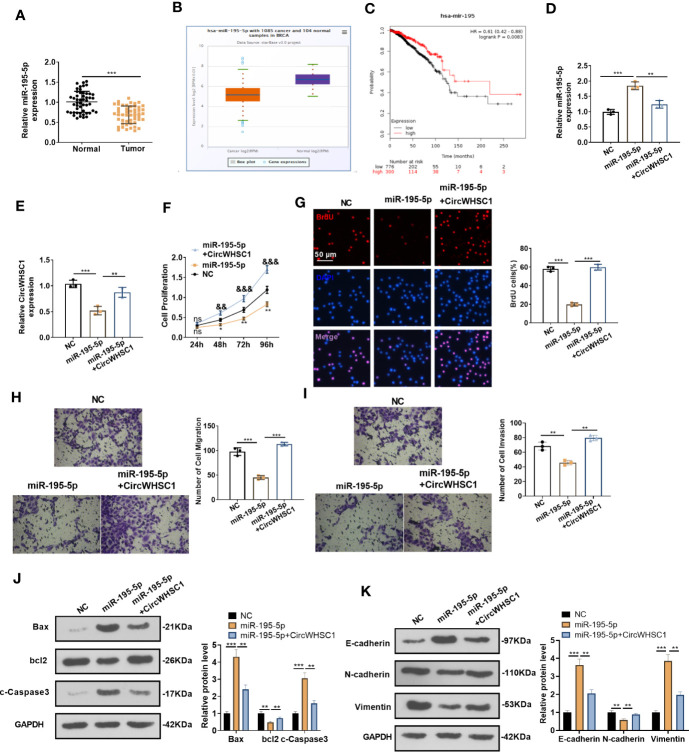
miR-195-5p inhibited BC progression and was dampened by circWHSC1. **(A)** The miR-195-5p profile in BC tissues was detected by qRT-PCR. **(B)** The expression of miR-195-5p in 1085 BC tissues and 104 non-tumor tissues was shown by Starbase. **(C)** Analysis of the link between miR-195 and BC prognosis *via* KMplotter database (http://kmplot.com/analysis/). **(D, E)** MCF7 cells were transfected with miR-195-5p mimics and circWHSC1 overexpressing plasmids, and qRT-PCR was carried out to determine the expression of miR-195-5p **(D)** and circWHSC1 **(E)**. **(F)** CCK-8 assay was used for detecting cell proliferation, **P < 0.05, **P < 0.01 (vs.NC group), ^&&^ *P < *0.01, ^&&&^ *P <* 0.001 (vs. miR-195-5p group). **(G)** BrdU assay evaluated cell proliferation. scale bar=50 μm. **(H, I)** Transwell assay determined MCF7 cell migration and invasion. **(J, K)**. WB was performed for evaluating the protein levels of Bax, bcl2, c-Caspase3, E-cadherin, N-cadherin, and Vimentin. **P < 0.01, ***P < 0.001. N=3. NS indicates **P < 0.01 (vs. NC group), NC is short for Negative control.

By detecting the expression of miR-195-5p in BC tissues, we concluded that the miR-195-5p level was significantly decreased in BC tissues versus non-tumor tissues (*P <*0.05, **Figure 5A**). Similar result was discovered by the data analysis on Starbase (**Figure 5B**). The lower level of miR-195 was linked to the worse overall survival of BC patients, as evidenced by KM plotter analysis (https://kmplot.com/analysis/index.) (**Figure 5C**). Considering that miR-195-5p was directly targeted by circWHSC1, we were interested in the contribution of the circWHSC1-miR-195-5p axis in BC. The co-transfection of circWHSC1 overexpression plasmids and miR-195-5p mimics showed that miR-195-5p was down-regulated and circWHSC1 was up-regulated in circWHSC1+miR-195-5p group versus the miR-195-5p group (**Figures 5D, E**). Next, we conducted CCK-8 and BrdU assay to test cell proliferation. It turned out that overexpressing miR-195-5p decreased cell proliferation, while upregulating circWHSC1 enhanced cell proliferation versus the miR-195-5p group (**Figures 5F, G**). Moreover, the Transwell assay revealed that miR-195-5p repressed the migrative and invasive capacities of BC cells. circWHSC1 transfection resulted in strengthened BC cell migration and invasion versus the miR-195-5p group (**Figures 5H, I**). We conducted WB for evaluating apoptosis- and EMT-related proteins and discovered that the miR-195-5p group had less bcl2, N-cadherin, and Vimentin, along with enhanced Bax, cleaved Caspase3, and E-cadherin expression. However, circWHSC1 overexpression plasmids’ transfection up-regulated bcl2, N-cadherin, and Vimentin and down-regulated Bax, cleaved Caspase3, and E-cadherin (**Figures 5J, K**). Taken together, miR-195-5p was a tumor suppressor in BC and circWHSC1 reversed miR-195-5p-mediated anti-tumor functions.

### FASN Was Targeted by miR-195-5p

**Figure 6:**
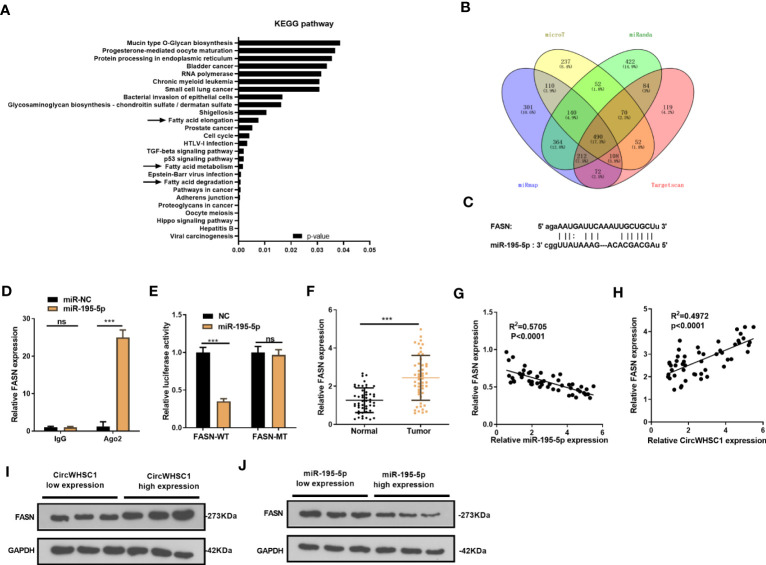
FASN was targeted by miR-195-5p. **(A)**. miRpath was employed to analyze the related pathways modulated by miR-195-5p. **(B)** Venn Diagram was utilized to investigate the candidate targets of miR-195-5p among four databases (including microT, Targetscan, miRanda and miRmap). **(C)** The binding sites of the 3’UTR of FASN mRNA with miR-195-5p. **(D)** RIP assay tested the targeting association between miR-195-5p and FASN. **(E)** The dual-luciferase reporter gene assay detected the binding of FASN with miR-195-5p. **(F)** qRT-PCR tested the FASN mRNA level in BC tissues. **(G, H)** Pearson linear regression analysis revealed that FASN mRNA was negatively related to miR-195-5p expression **(G)** and positively correlated with the circWHSC1 level **(H)**. **(I, J)** WB was conducted to detect FASN in BC tissues with different levels of circWHSC1 **(I)** or miR-195-5p **(J)**. ns *P* > 0.05, ***P < 0.001. N=3.

We were curious about the downstream mechanism of miR-195-5p in BC. Through miRpath (http://snf-515788.vm.okeanos.grnet.gr/index.php?r=miRpath) analysis, we believed that miR-195-5p is effective in modulating the fatty acid-related pathways (**Figure 6A**). Next, we analyzed the target genes regulated by miR-195-5p *via* Starbase and validated that FASN is a candidate (**Figures 6B, C**). To test the targeting association between miR-195-5p and FASN, we performed RIP assay. It turned out that enriched FSAN level was significantly higher in the Ago2 antibody group versus the IgG group after miR-195-5p transfection, indicating FASN bound to Ago2 *via* miR-195-5p (*P <*0.05, **Figure 6D**). We performed dual-luciferase reporter gene assay, and found that miR-195-5p greatly decreased the luciferase activity of FASN-WT-transfected cells, while it had little influence on that of FASN-MT (*P*> 0.05, **Figure 6E**). As shown by qRT-PCR result, FASN was markedly up-regulated versus that in adjacent non-tumor tissue*s* (*P <*0.05, **Figure 6F**). Furthermore, Pearson linear regression analysis disclosed that FASN had a negative relationship with miR-195-5p in BC tissues (*P <*0.0001, *R* ^2^ = 0.5705, **Figure 6G**). In contrast, FASN and circWHSC1 levels were positively correlated in BC tissues (*P <*0.0001, *R* ^2^ = 0.4972, **Figure 6H**). We detected FASN levels at low or high levels of circWHSC1 and miR-195-5p. WB results showed that FASN had higher expression in BC tissues with high CircWHSC1 expression and low miR-195-5p expression (**Figures 6I, J**). These data indicated that FASN acts as a downstream target of miR-195-5p, and there is a potential circWHSC1/miR-195-5p/FASN axis in BC.

### The Modulation of circWHSC1 and miR-195-5p on FASN

**Figure 7:**
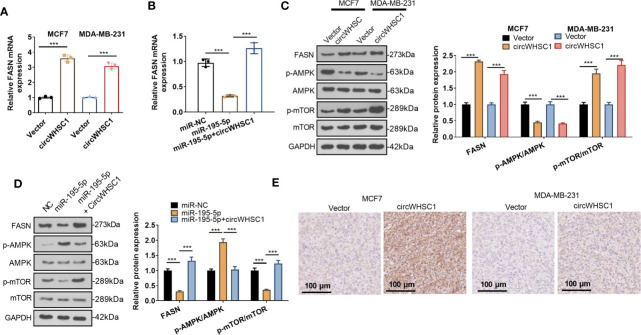
CircWHSC1 and miR-195-5p regulated FASN. **(A, B)** qRT-PCR examined FASN mRNA expression in BC cells transfected with circWHSC1 overexpressing plasmids or miR-195-5p mimics. **(C, D)** WB tested FASN, AMPK and mTOR levels. **(E)**. IHC evaluated FASN in the tumor tissues formed by MCF7 and MDA-MB-231 cells transfected with vector or circWHSC1 overexpression plasmids. scale bar=100 μm. ***P < 0.001. N=3.

For making certain the circWHSC1/miR-195-5p/FASN pathway in BC, the FASN mRNA and protein contents were evaluated by using qRT-PCR and WB, respectively. We found that circWHSC1 overexpression elevated FASN expression, while miR-195-5p upregulation inhibited FASN expression (**Figures 7A**–**D**). In addition, the AMPK/mTOR pathway was detected. It was observed that overexpressing circWHSC1 markedly decreased p-AMPK level, and facilitated FASN and p-mTOR levels (**Figure 7C**). By contrast, overexpressing miR-195-5p up-regulated p-AMPK and down-regulated p-mTOR. However, up-regulating circWHSC1 in the miR-195-5p overexpressing group led to down-regulated p-AMPK and up-regulated FASN and p-mTOR versus the miR-195-5p group (*P <*0.05, **Figure 7D**). IHC was conducted for evaluating FASN in the formed tumor tissues as shown in **Figure 3**. FASN level in the circWHSC1 overexpressing group was enhanced versus the vector group (**Figure 7E**). These results displayed that circWHSC1 affected the FASN/AMPK/mTOR expression by regulating miR-195-5p.

### Attenuation of FASN or Activation of AMPK Reversed circWHSC1-Mediated Pro-Carcinogenic Effects

**Figure 8:**
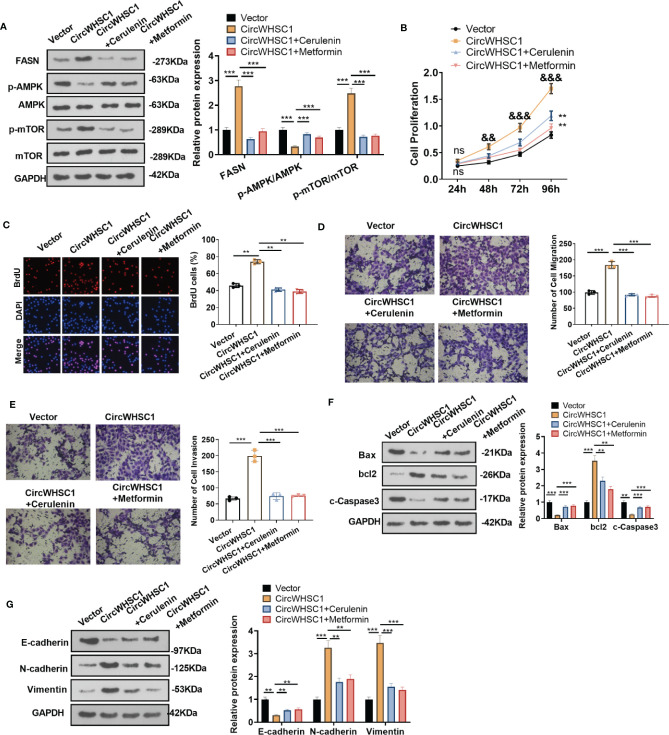
Inhibiting FASN or activating the AMPK pathway reversed circWHSC1-mediated oncogenic effects on BC cells. MCF7 cells were transfected with the negative vector or circWHSC1 overexpression plasmids and then treated with the FASN inhibitor Cerulenin (2 mg/L) or the AMPK activator Metformin (20 mM). **(A)** WB tested FASN, AMPK and mTOR levels. **(B)** CCK-8 tested cell viability. ns *P* > 0.05, **P < 0.01 (vs.vector group), ^&&^ *P <* 0.01, ^&&&^ *P <* 0.001 (vs. circWHSC1 group). **(C)**. BrdU staining evaluated cell proliferation. **(D, E)** Cell migration and invasion were tested by the Transwell assay. **(F, G)** WB assessed the expressions of Bax, bcl2, and c-Caspase3 and EMT-related proteins E-cadherin, N-cadherin and Vimentin. **P < 0.01, ***P < 0.001. N=3.

**Figure 9:**
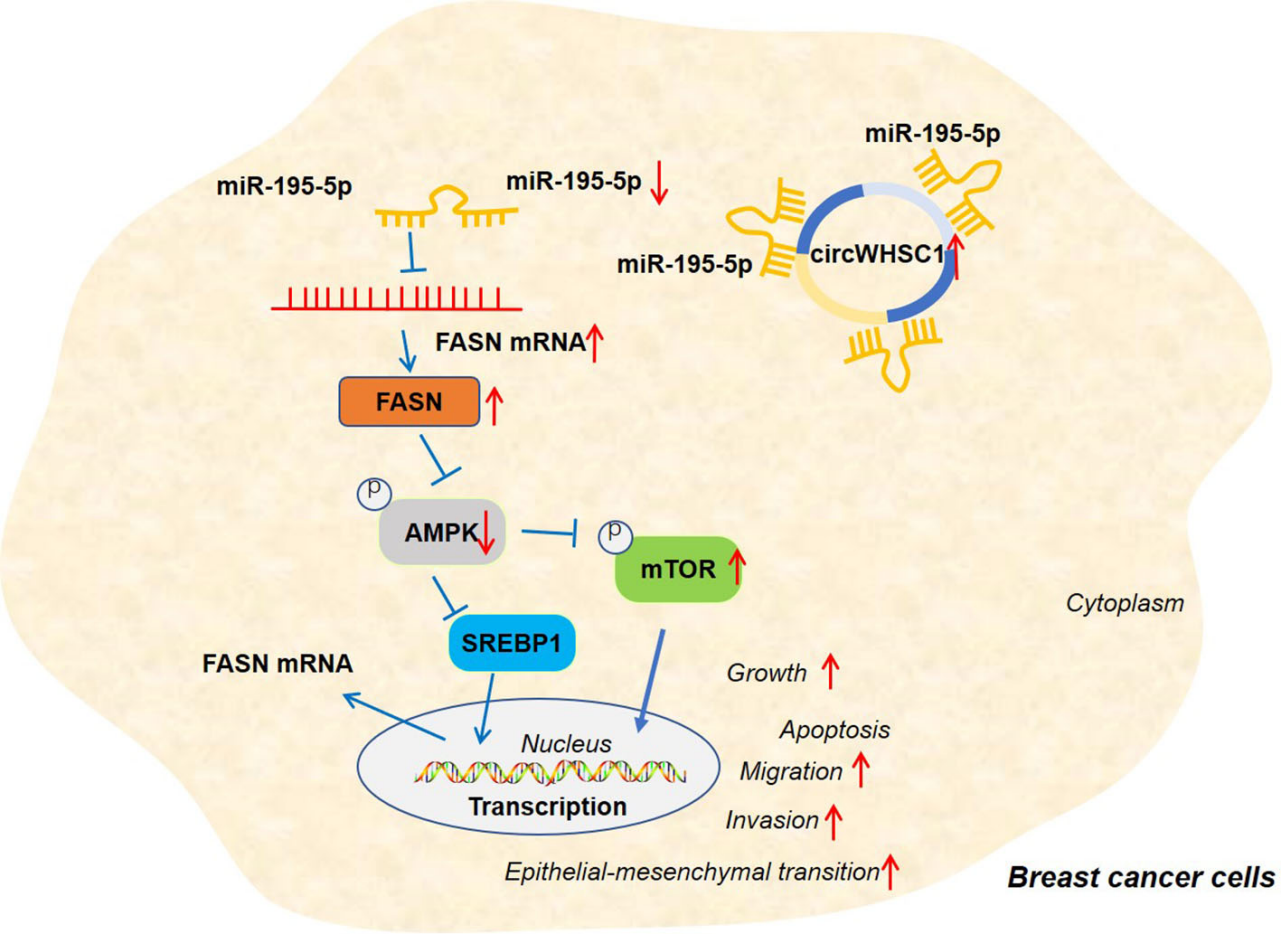
The graphical abstract of the circWHSC1-miR-195-5p-FASN/AMPK/mTOR axis on BC development. circWHSC1 is up-regulated in BC. CircWHSC1 targets miR-195-5p, which inhibits FASN expression. FASN induces inhibitive effects on the AMPK pathway, thus enhancing proliferation, growth, migration, invasion, EMT and relieving apoptosis.

To confirm the mechanism of FASN/AMPK pathway in circWHSC1-mediated carcinogenic effects, we treated MCF7 cells with the FASN inhibitor Cerulenin or the AMPK activator Metformin. The expression of FASN and the AMPK-mTOR pathway was checked by WB. Notably, in comparison to the circWHSC1 group, Cerulenin and Metformin treatment resulted in FASN and mTOR phosphorylation down-regulation, and dramatic facilitation in AMPK phosphorylation level (**Figure 8A**). Next, we conducted the CCK-8 assay and BrdU assay. Cerulenin and Metformin treatment caused a significant reduction in MCF7 viability and proliferation versus the circWHSC1 group (**Figures 8B, C**). As evidenced by the Transwell assay, the application of Cerulenin and Metformin substantially attenuated the migratory and invasive ability of MCF7 cells versus the circWHSC1 group (**Figures 8D, E**). WB was carried out to evaluate the apoptosis-associated proteins and EMT-associated proteins. The data revealed that Cerulenin and Metformin treatments up-regulated Bax, c-Caspase3, and E-cadherin, and reduced the levels of bcl2, N-cadherin and Vimentin (vs. circWHSC1 group, **Figures 8F, G**). Thus, circWHSC1 exerts carcinogenic effects mainly through regulating the FASN-AMPK axis (**Figure 9**).

## Discussion

Emerging circRNAs are found to play a role in BC (18). Therefore, studying the biological function of circRNAs and their potential mechanisms makes sense in the clinical treatment and diagnosis of BC. Here, we found that circWHSC1 was up-regulated in BC tissues and acted as a potential prognostic biomarker for BC patients.

CircRNAs are a special type of noncoding RNAs, which modulate tumor-related gene expression and participate in tumorigenesis as a ceRNA (19, 20). For example, circACAP2 is up-regulated in BC tissues, which elevates cell growth and metastasis by controlling the miR-29A/B-3P/COL5A1 axis (21). Zhang Wei et al. reported that circHOXC-AS3 up-regulates PPP1R1A by sponging miR-3922-5p, thus promoting BC cell growth and metastasis (22). Besides, circRNAs modulate the chemotherapy sensitivity of BC cells. As an example, circ_0025202 hampers BC cells’ proliferation, colony formation, and migration and enhances their apoptosis and sensitivity to tamoxifen (23). circWHSC1 functions as an oncogene in multiple tumors, including ovarian cancer (9), endometrial cancer (24), and hepatocellular carcinoma (25). In terms of its functions, circWHSC1 increases tumor cell proliferation, migration and invasion, and suppresses cell apoptosis. Here, we concluded that circWHSC1 functions as an unfavorable biomarker in BC patients, and overexpressing circWHSC1 remarkably accelerates BC cells’ proliferation, reduces apoptosis and promotes metastasis, indicating that circWHSC1 is an oncogene in BC.

miRNAs have been a research hotspot in the field of oncology, including BC (26, 27). miRNAs are dysregulated during BC progression, and several miRNAs serve as survival and prognosis biomarkers (28, 29). Remarkedly, miRNAs modulate the proliferation, metastasis, chemosensitivity, apoptosis, and other malignant phenotypes (30). For example, miR-532-5p is overexpressed in BC tissues. It reduces RERG expression and activates the MAPK/ERK signaling to facilitate BC cell growth and migration (31). Besides, miR-302b is obviously downregulated in BC tissues and cell lines, and overexpressing miR-302b dramatically attenuates the growth and metastasis of BC cells (BT549 and MCF-7) (32). Moreover, miR-195-5p modulates the evolvement of BC. For instance, miR-195-5p is down-regulated in 40 BC specimens versus adjacent normal breast tissues. Overexpressing miR-195-5p notably represses BC cell proliferation, migration, and invasion by dampening CCNE1 expression (33). Consistent with earlier reports, miR-195-5p is found down-regulated in BC in this study Forced miR-195-5p up-regulation significantly declines cell proliferation and metastasis, confirming the tumor-repressive role of miR-195-5p in BC.

As reported, circRNAs function by sponging their target miRNAs. When more circRNAs are expressed in tumor cells, their targeted miRNAs become inactivated or degraded, resulting in lower miRNA levels in the cytoplasm (34, 35). For example, Yang Rui et al. claimed that circAGFG1 accelerates BC cell proliferation and migration by sponging miR-195-5p (36). Presently, we found that circWHSC1 level was reversely related to miR-195-5p level in BC tissues. By conducting RIP and dual-luciferase reporter assay, we confirmed that circWHSC1 bound to miR-195-5p and inhibited its expression. Functionally, overexpressing circWHSC1 reversed the anti-tumor effects caused by miR-195-5p, which partly explained how circWHSC1 exerted its promotive effects on the development of BC.

Fatty acid metabolism dysregulation has been regarded as a component of malignant transformation in BC (37). FASN is a multifunctional enzyme responsible for endogenous fatty acids synthesis, which is up-regulated in various human malignancies and related to poor prognosis (38). SPIN1 triggers FASN signals through SREBP1c to regulate abnormal liver lipid metabolism and promote liver cancer cell growth (39). More impressively, FASN inhibition enhances the sensitiveness of tumor cells to chemotherapy (40) and radiotherapy (41). Those studies indicate that FASN acts as an oncogene in diversified tumors. In addition, FASN, an important functional target of miR-127, also makes a great contribution to BC. miR-127 targets FASN to reduce the viability and motility of triple-negative BC cells (42). In this study, the bioinformatic analysis showed that miR-195-5p had binding sites with FASN. Further experiments exhibited that FASN was obviously overexpressed in BC and was reversely related to miR-195-5p. circWHSC1 was positively linked to FASN expression, and circWHSC1 overexpression significantly increased FASN expression in BC. Hence, circWHSC1 could promote BC evolvement by modulating the miR-195-5p/FASN axis.

Protein kinase AMP-activated catalytic subunit alpha 1 (AMPK) is a cellular energy transducer that is conserved in all eukaryotic cells. It controls the activities of various key metabolic enzymes *via* phosphorylation modification (43). On the other hand, the mechanistic target of rapamycin kinase (mTOR) belongs to the phosphatidylinositol kinase-related kinase family. These kinases coordinate cellular responses to stresses such as DNA injury and nutrient insufficiency (44). During cancer development, AMPK/mTOR pathway becomes dysregulated (45). What is more, FASN enhances CRC cells’ proliferation and metastasis by attenuating the AMPK/mTOR pathway activation (46). Here, we observed that overexpressing miR-195-5p increased AMPK phosphorylation and dampened mTOR phosphorylation. Overexpressing circWHSC1 had the opposite effects. Meanwhile, the influence of miR-195-5p on the AMPK/mTOR axis activation was mostly altered by circWHSC1 up-regulation. We treated BC cells with the FASN inhibitor Cerulenin (47) or the AMPK agonist Metformin (48). The proliferation, invasion and migration of BC cells with circWHSC1 overexpression were notably inhibited. Considering that circWHSC1/miR-195-5p had obvious effects on modulating FASN expression, we believed that circWHSC1 regulated the FASN/AMPK/mTOR axis by modulating miR-195-5p expression.

Overall, our research confirms that circWHSC1 is a good prognostic biomarker for BC. In addition, circWHSC1 promotes BC cell proliferation and metastasis as a therapeutic target for BC. Therefore, it is believed that there is a novel circWHSC1-miR-195-5p-FASN/AMPK/mTOR axis in BC (**Figure 9**). This study offers a better insight into the molecular mechanism of BC, which is helpful for BC’s early diagnosis and treatment.

## Data Availability Statement

The datasets presented in this study can be found in online repositories. The names of the repository/repositories and accession number(s) can be found in the article/supplementary material.

## Ethics Statement

The studies involving human participants were reviewed and approved by the Ethics Review Board of Minhang Hospital, Fudan University. The patients/participants provided their written informed consent to participate in this study. The animal study was reviewed and approved by The Ethics Review Board of Minhang Hospital, Fudan University. Written informed consent was obtained from the individual(s) for the publication of any potentially identifiable images or data included in this article.

## Author Contributions

Conceived and designed the experiments: WW and ZP. Performed the experiments: QC and WW. Statistical analysis: ZY, HD, and HL. Wrote the paper: QC and ZP. All authors contributed to the article and approved the submitted version.

## Conflict of Interest

The authors declare that the research was conducted in the absence of any commercial or financial relationships that could be construed as a potential conflict of interest.

## Publisher’s Note

All claims expressed in this article are solely those of the authors and do not necessarily represent those of their affiliated organizations, or those of the publisher, the editors and the reviewers. Any product that may be evaluated in this article, or claim that may be made by its manufacturer, is not guaranteed or endorsed by the publisher.
